# Ultrasonographic caval indices do not significantly contribute to predicting fluid responsiveness immediately after coronary artery bypass grafting when compared to passive leg raising

**DOI:** 10.1186/s12947-016-0065-4

**Published:** 2016-06-08

**Authors:** Dorota Sobczyk, Krzysztof Nycz, Pawel Andruszkiewicz, Karol Wierzbicki, Maciej Stapor

**Affiliations:** 1 Department of Interventional Cardiology, John Paul II Hospital, Cracow, Poland; 2 Emergency and Admission Department, John Paul II Hospital, Pradnicka 80, 31 202 Cracow, Poland; 3 2nd Department of Anaesthesiology and Intensive Care, Warsaw Medical University, Warsaw, Poland; 4 Cardiovascular Surgery and Transplantology Department, Medical College, Jagiellonian University, Cracow, Poland

**Keywords:** Ultrasound-guided fluid therapy, Coronary artery bypass grafting, Inferior vena cava, Caval index, Fluid responsiveness, Passive leg raising, Fluid challenge

## Abstract

**Background:**

Appropriate fluid management is one of the most important elements of early goal-directed therapy after cardiothoracic surgery. Reliable determination of fluid responsivenss remains the fundamental issue in volume therapy.

The purpose of the study was to assess the usefulness of dynamic IVC-derived parameters (collapsibility index, distensibility index) in comparison to passive leg raising, in postoperative fluid management in mechanically ventilated patients with left ventricular ejection fraction ≥ 30 %, immediately after elective coronary artery bypass grafting.

**Methods:**

Prospective observational case series study including 35 patients with LVEF ≥ 30 %, undergoingelective coronary artery bypass grafting was conducted. Transthoracic echocardiography, passive leg raising and intravenous administration of saline were performed in all study subjects. Dynamic parameters derived from ultrasonographic assessment of the IVC diameter (collapsibility index–CI and distensibility index–DI), cardiac output

**Results:**

There were 24 (68.57 %) responders in the study population. There were no statistical differences between the groups in relation to: clinical parameters, pre- and postoperative LVEF, fluid balance and CVP. Change in cardiac output after passive leg raising correlated significantly with that after the volume expansion (p=0.000, r=0.822). Dynamic IVC derivatives were slightly higher in fluid responders, however this trend did not reach statistical significance. None of the caval indices correlated with fluid responsiveness.

**Conclusion:**

Dynamic IVC-derived parameters do not predict fluid responsiveness in mechanically ventilated patients with preserved ejection fraction immediately after elective coronary artery bypass grafting. Passive leg raising is not inferior to volume expansion in differentiating between fluid responders and nonresponders. Immediate fluid challenge after CABG is safe and well tolerated.

**Electronic supplementary material:**

The online version of this article (doi:10.1186/s12947-016-0065-4) contains supplementary material, which is available to authorized users.

## Background

Postoperative goal-directed therapy has been shown to substantially improve clinical outcomes in surgical patients [1, 2]. Two important aspects of a goal-directed protocol include definition of hemodynamic target and early initiation of the therapeutic measures [3, 4]. Appropriate fluid management is one of the most important elements of early goal-directed therapy after cardiothoracic surgery [5–7]. Therefore, the fundamental issue in a goal-directed volume therapy remains determination of the potential response to fluid challenge, e.g. fluid responsiveness [8, 9].

Among the numerous monitoring methods, ultrasonographic measurement of inferior vena cava (IVC) diameter respiratory variation seems to meet the criteria of an ideal bedside tool to assess the individual fluid responsiveness. However, this method has been validated in selected populations (e.g. hemodialysis, septic shock, subarachnoid hemorrhage) or healthy volunteers, and its usefulness has not been confirmed in patients after cardiac surgery [10–15]. In our recent study, IVC-derived dynamic indices failed to predict fluid responsiveness in the first six hours after cardiothoracic procedures [16]. Nevertheless, heterogeneity of the study population (different cardiac surgery procedures, concomitant valve disease) have accounted to the study results. Usefulness of ultrasonographic measurement of IVC respiratory variation in selected cardiosurgical patients (e.g. coronary artery bypass grafting, preserved left ventricular systolic function) remains still an open question.

The purpose of the present study was to assess the usefulness of dynamic IVC-derived parameters (collapsibility index, distensibility index) in comparison to passive leg raising, in postoperative fluid management in mechanically ventilated patients with left ventricular ejection fraction ≥30 %, immediately after elective coronary artery bypass grafting.

## Methods

### Study population and methods

The study population consisted of 35 consecutive adult patients admitted to our hospital for elective coronary artery bypass grafting. Exclusion criteria were: age <18 years, preoperative left ventricular ejection fraction (LVEF) <30 %, preoperative left ventricular dilatation (end-diastolic dimension ≥6 cm), preoperative severe tricuspid valve regurgitation, preoperative right ventricular dysfunction (tricuspid annular plane systolic excursion <16 mm), at least moderate aortic valve disease, at least moderate mitral valve disease and difficult acoustic window resulting in inability to obtain interpretable ultrasound images. Coronary artery bypass grafting was performed using cardiopulmonary bypass. In all patients full midline sternotomy was made. Mediastinal drainage (midline drain tubes) was placed in all patients. Additionally, those with arterial graft with left internal mammary artery had a drain placed in left pleural cavity. All patients were operated in moderate hypothermia (32–33 °C) and warmed up to 36.6 °C within two hours following the end of the operation. Total intravenous anesthesia with propofol, sufentanil and pancuronium was used during the procedure. Propofol infusion was continued for 1-hour in the ICU and morphine infusion was used for postoperative pain relief. Cardiac ultrasound was performed when the patients were ventilated (SIMV-mode, tidal volume: 8 ml/kg, PEEP: 4.5 cmH_2_O).

The following baseline data were recorded for each patient: age (years), gender, weight (kg), height (cm), number of bypassed vessels, aortic cross-clamping duration, cardiopulmonary bypass duration, and preoperative echocardiographic parameters (left ventricular ejection fraction, presence of left ventricular hypertrophy, right ventricular end-diastolic diameter and tricuspid regurgitation grade). Pulse pressure was calculated as the difference between systolic and diastolic pressure readings (expressed in mmHg). The use of the following vasoactive drugs was noted: nitroglycerine, dopamine, dobutamine, epinephrine, norepinephrine.

Transthoracic bedside echocardiography was performed by two trained investigators, both with at least 5 years of experience in emergency ultrasound. The examinations were conducted with portable ultrasound system equipped with a 1–5 MHz transthoracic phased-array transducer (CX 50 Philips, Eindhoven, Netherlands). All the studies were recorded as digital clips and reviewed independently by both sonohraphers.

Inferior vena cava was visualized longitudinally in the subcostal view. Maximal and minimal IVC diameters (IVCmax and IVCmin, retrospectively) were measured using 2D image, distally to the hepatic vein inlet, over a single respiratory cycle. A total of three measurements were obtained and averaged for each IVC diameter. The IVC collapsibility index (IVC-CI) was defined as: IVC-CI = IVCmax - IVCmin/IVCmax. The IVC distensibility index (IVC-DI) was calculated using the formula: IVC-DI = IVCmax - IVCmin/IVCmin. Both indices were expressed as a percentage.

Cardiac output (CO) was calculated from the left ventricular outflow tract (LVOT), using the previously described equation [17]: CO = 0.785 x dLVOT^2^ x VTI LVOT x HR. The left ventricular outflow tract diameter (dLVOT) was measured in midsystole, in a parasternal long-axis view immediately adjacent to the aortic valve. LVOT velocity time integral (LVOT VTI) was recorded by pulsed Doppler imaging from a three-chamber or five-chamber apical view. Left ventricular ejection fraction (LVEF) was assessed by visual inspection.

### Study protocol

Hemodynamic and ultrasound data were obtained at four sequential steps per enrollment: (1) at baseline, immediately after cardiac surgery procedure, when the patient was transferred to intensive care unit, in the semirecumbent position (45 °); (2) during passive leg raising (after 1 min), when the patient’s lower limbs were raised to a 45 ° angle while the patient’s trunk was lowered in supine position; (3) in the semirecumbent position (as a baseline data for the next stage); (4) after a 10-min infusion of 250 ml of saline, in the semirecumbent position. Fluid balance immediately after cardiac surgery was noted. The following parameters were recorded at each study step: heart rate (HR, bmp), systolic blood pressure (SPB, mmHg), diastolic blood pressure (DBP, mmHg), central venous pressure (CVP, mmHg), maximal (IVCmax, mm) and minimal (IVCmin, mm) diameters of inferior vena cava, left ventricular ejection fraction (LVEF, %), velocity time integral in left ventricular outflow tract (VTI LVOT, cm). Fluid responsiveness was defined as an increase in cardiac output ≥15 % after the fluid challenge that defined patients as responders and non-responders. Any clinically significant findings (e.g. severe left ventricular dysfunction, cardiac tamponade) were immediately reported to the treating physician.

### Statistical analysis

Statistical analysis was performed using STATISTICA v 8.0 software. The required size of the study group was determined with power analysis. Using prior estimates of expected correlation, a sample size of 9 for each subgroup (responders and nonresponders) was determined, with α = 0.05 and β = 0.8. Numerical data were expressed as mean values ± SD. After determining the probability distribution with Shapiro-Wilk test, the comparisons between fluid responders and nonresponders were performed with U Mann–Whitney test. A p-value of 0.05 was considered significant. The correlations between different parameters were evaluated with r-Spearman correlation analysis. Scatterplots for the two variables were drawn with confidence interval 0.95. The area under the receiver operator characteristics curve (AUROC) was used to determine the diagnostic accuracy of the methods.

## Results

**Fig. 1:**
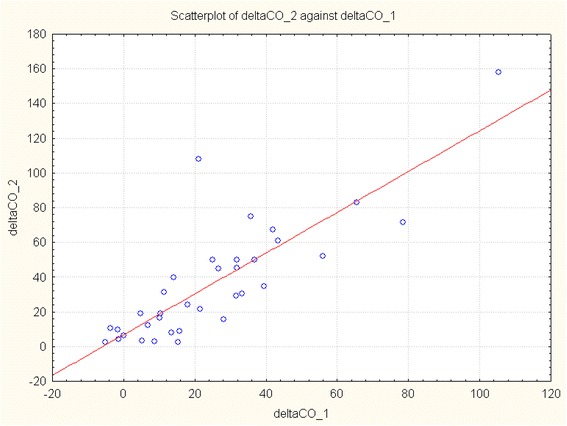
Scatterplot showing the correlation between change in cardiac output after passive leg raising (ΔCO_1) and that induced by volume expansion (ΔCO_2) (*p* = 0.000, *r* = 0.822)

**Fig. 2:**
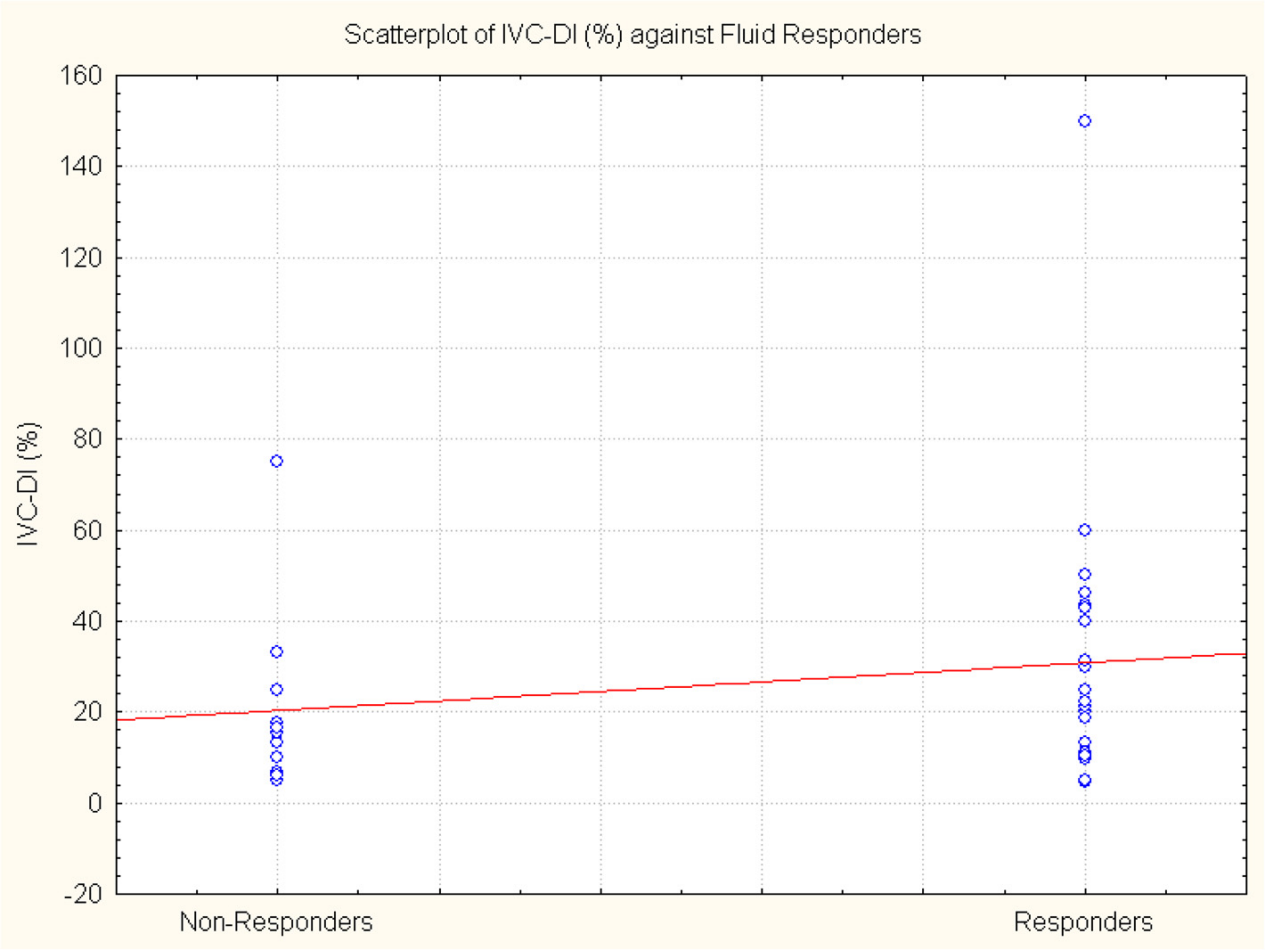
Scatterplot showing the distribution of IVC-DI values in fluid responders and nonresponders

The study population consisted of 35 consecutive patients (25 male, age 51–81 years, median 65 years) who underwent elective coronary artery bypass grafting Additional file 1. Baseline demographic and clinical data are summarized in Table 1. There were 1–4 bypassed vessels (median 3 bypasses), in 24 patients (80 %) left internal mammary artery was used for arterial grafts. All patients were in sinus rhythm. Fluid balance immediately after cardiac surgery was positive in the overall study population (150–2350 ml, median 1000 ml). Patients were divided into fluid responders and nonresponders based on a ≥15 % increase in cardiac output after the infusion of 250 ml of saline. There were 24 (68.57 %) responders and 11 nonresponders in the study population. Table 2 provides baseline clinical characteristics in responders and nonresponders. Table 3 shows hemodynamic parameters in these patients on different stages of the study protocol. There were no statistical differences between both groups in relation to: clinical parameters (HR, SBP, DBP, MAP, PP), pre- and postoperative LVEF, postoperative fluid balance and CVP. Neither passive leg raising nor intravenous fluid challenge influenced significantly heart rate, blood pressure (SBP, DBP, MAP, PP) and IVC-derived parameters. Change in cardiac output after passive leg raising (ΔCO_1) correlated well with that induced by volume expansion (ΔCO_2) (*p* = 0.000, *r* = 0.822) (Fig. 1). A 15 % PLR-induced increase of cardiac output predicted a 15 % increase in cardiac output after fluid administration, with 79.17 % sensitivity and 81.82 % specificity. The diagnostic accuracy of PLR (area under ROC curve) was 0.805. Both dynamic IVC derivatives (IVC-CI, IVC-DI) were slightly higher in fluid responders when compared to nonresponders, however this trend did not reach statistical significance (Fig. 2). Furthermore, none of the caval indices correlated with change in cardiac output after fluid challenge (*p* = 0.357 and *r* = 0.16 for IVC-CI; *p* = 0.358 and *r* = 0.16 for IVC-DI). The general diagnostic accuracy of IVC-DI (AUROC) was 0.647. When using the value of 18 % (according to the literature) as a cut-off point distinguishing responders form nonresponders, the AUROC increased to 0.739. IVC-DI of ≥18 % had sensitivity of 82.35 % and specificity of 72.72 % in predicting a 15 % increase in cardiac output after fluid administration.

**Table 1:** Demographic and clinical characteristics of the study population

Characteristic	
Age (yrs)	66.66 ± 8.39
Male sex, n (%)	25 (71.43)
Body mass index (kg/m^2^)	27.91 ± 4.83
Left ventricular ejection fraction (%)	46.89 ± 11.9
Left ventricular hypertrophy, n (%)	13 (37.14)
Right ventricular end-diastolic dimension (mm)	31.74 ± 4.83
Tricuspid regurgitation, n (%)	35 (100)
Trace	23 (65.71)
Mild	8 (22.86)
Moderate	4 (11.43)
Number of bypassed coronary arteries	2.5 ± 0.66
Aortic cross-clamping time (min.)	44.89 ± 13.98
Cardiopulmonary bypass time (min.)	86.09 ± 26.66
Vasoactive drugs, n (%)	30 (85.71)
Baseline fluid balance (ml)	1200.29 ± 745.88

**Table 2:** Baseline hemodynamic parameters in fluid responders and nonresponders

Parameter	Mean value ± SD (median)
Preoperative LVEF (%)	
Responders	45.71 ± 12.79 (46)
Nonresponders	49.45 ± 9.71 (50)
Fluid balance (ml)	
Responders	1227.5 ± 810.79 (1025)
Nonresponders	1140.91 ± 611.48 (1000)
Postoperative LVEF (%)	
Responders	36.04 ± 11.7 (35)
Nonresponders	41.36 ± 12.47 (40)
Use of vasoactive drugs (%)	
Responders	60
Nonresponders	81.82
CVP (mmHg)	
Responders	5.04 ± 2.22 (5)
Nonresponders	6.72 ± 2.61 (6)
IVC-CI (%)	
Responders	21.08 ± 17.9 (17.9)
Nonresponders	15.28 ± 11.04 (13.3)
IVC-DI (%)	
Responders	30.85 ± 21.8 (21.8)
Nonresponders	20.35 ± 20.02 (15.4)

*Abbreviations: SD* standard deviations, *LVEF* left ventricular ejection fraction, *CVP* central venous pressure, *IVC-CI* inferior vena cava collapsibility index, *IVC-DI* inferior vena cava distensibility index

**Table 3:** Hemodynamic parameters in responders and nonresponders in different phases of the study

Parameter	Baseline	PLR	Fluid challenge
mean ± SD (median)			
HR (bpm)			
Responders	80.63 ± 11.33 (80)	80.88 ± 12.28 (81)	81.5 ± 12.3 (82.5)
Nonresponders	84 ± 11.74 (82)	84.45 ± 10.54 (84)	84.91 ± 10.36 (84)
SBP (mmHg)			
Responders	109.54 ± 23.99 (107)	115.75 ± 20.36 (117.5)	113.7 ± 16.76 (114)
Nonresponders	115.72 ± 20.07 (119)	123.09 ± 22.72 (128)	124.55 ± 21.94 (126)
DBP (mmHg)			
Responders	59.63 ± 13.28 (60)	58.17 ± 10.45 (58.5)	57.79 ± 11.82 (59.5)
Nonresponders	58.91 ± 9.44 (60)	62.73 ± 9.96 (64)	62.27 ± 12.85 (67)
MAP (mmHg)			
Responders	74.88 ± 15.59 (72.5)	77.25 ± 12 (76)	75.88 ± 12.84 (76.5)
Nonresponders	77.64 ± 12.73 (77)	83.55 ± 14.51 (83)	84.27 ± 16.22 (84)
PP (mmHg)			
Responders	49.67 ± 19.57 (48.5)	57.58 ± 18.64 (58)	55.92 ± 13.95 (56)
Nonresponders	56.82 ± 15.53 (61)	60.36 ± 16.68 (65)	59.27 ± 13.68 (62)

*Abbreviations: HR* heart rate, *SBP* systolic blood pressure, *DBP* diastolic blood pressure, *MAP* mean blood pressure, *PP* pulse pressure, *SD* standard deviation

## Discussion

This prospective observational case series was designed to test the effectiveness of IVC-derived parameters in predicting fluid responsiveness immediately after elective coronary artery bypass grafting. Our previous study did not confirm the utility of caval indices as a guiding tool for early postoperative fluid therapy in a heterogeneous group after cardiac surgery [16]. The present study was limited to patients qualified for elective coronary artery bypass surgery. To reduce any potential bias, severely diminished left ventricular ejection fraction (LVEF <30 %), LV dilatation, at least moderate aortic or mitral valve disease, impaired right ventricular systolic function (TAPSE <16 mm) and severe tricuspid regurgitation were the exclusion criteria from the study. All patients were mechanically ventilated with standard parameters to limit influence of spontaneous breathing on IVC diameter. The study protocol assumed cardiac output response (increase of ≥15 % compared to the baseline) after intravenous administration of 250 ml of saline as a reference to assess fluid responsiveness. Additionally, passive leg raising was used as an alternative reversible volume challenge in all patients.

The majority of patients in our study (almost 70 %) responded to fluid challenge with significant cardiac output increase and met the criteria of fluid responsiveness. While we excluded patients with severely impaired left ventricular systolic function and LV dilatation, the main contributor of stroke volume increase were preload conditions. It seems that response to fluid challenge was mainly determined by absolute (preoperative fluid restriction, intraoperative blood loss) and relative hypovolemia (increased vascular bed capacity induced by hypothermia, vasoactive agents and anesthetic drugs, as well as fluid shifts). In our study fluid challenge with 250 ml of saline/PLR was safe and well tolerated in all subjects. Considering our results, we assume that immediate administration of crystalloids directly after CABG regardless hemodynamic parameters is reasonable, low-risk profile management.

Ultrasonographic measurement of IVC respiratory variation did not reach statistical significance in differentiation between fluid responders and nonresponders. Although we observed a trend towards higher values of both IVC indices (IVC-CI and IVC-DI) in responder group, none of them was statistically significant. In our study material we observed high variation of recorded measurements with overlapping series of values. Thus, it was impossible to determine a cut-off point separating responders and nonresponders.

Ultrasonographic assessment of the inferior vena cava diameter has been proposed as a bedside, noninvasive marker of fluid status [10]. The usefulness of this method has been validated during hemodialysis and continuous ultrafiltration or in patients with septic shock [11–14]. IVC distensibility index has been proved to predict fluid responsiveness in mechanically ventilated patients with septic shock and subarachnoid hemorrhage (with cut-off value of 18 %) [10, 15]. However, conflicting results of several prospective studies in unselected patients indicated the need for a cautionary use of IVC ultrasound in everyday ICU/emergency practice. Muller et al. [18] revealed that in spontaneously breathing patients with acute circulatory failure, high IVC-CI values (>40 %) were usually associated with fluid responsiveness, however values <40 % did not exclude positive response to volume expansion. Corl et al. [19] did not confirm utility of IVC-CI in predicting fluid responsiveness in their heterogeneous sample of adult emergency department patients. Juhl-Olsen et al. showed no correlation between IVC-CI and magnitude of hemodynamic response to early hemorrhage in healthy blood donors [20].

There are multiple possible reasons contributing to limited usefulness of dynamic IVC derivatives in early postoperative period after CABG [21]. First of all, there are numerous factors influencing IVC diameter, its respiratory variation and vascular bed capacity, such as cardiopulmonary bypass, intraoperative hypothermia, anesthetics and vasoactive agents. Also surgical chest opening and postoperative suction drainage (with negative pressure of 15–20 cmH_2_O) may contribute to negative results of this paper. However very attractive, ultrasonographic definition of fluid responsiveness using IVC respiratory variation failed in early postoperative period after cardiothoracic surgery that was also described in our previous work [16].

Several diagnostic approaches have been tested to determine fluid responsiveness in mechanically ventilated patients. Respiration-induced changes in arterial pulse pressure (ΔPP), based on the variation in arterial pressure associated with mechanical ventilation, have been demonstrated to accurately predict fluid responsiveness in mechanically ventilated patients with regular heart rhythm, who are making no respiratory efforts [22]. However, ΔPP is of poor value to predict fluid responsiveness in patients triggering the ventilator or in the presence of arrhythmias [23]. Additionally, reliable calculation of ΔPP is time-consuming, requires experience and specialized computer software. Therefore, this method is not commonly used for navigating the fluid therapy in the intensive care unit. Several publications have proposed using PLR maneuver to predict preload responsiveness [24]. Passive leg raising transfers about 300 ml of blood from the lower limbs to the intrathoracic compartment, mimicking fluid expansion [24–26]. PLR maneuver is quick, easy to perform at the bedside and completely reversible (with no side effects connected with actual fluid challenge). Proper PLR performance is essential for its reliability. Monnet and Teboul recently described 5-step protocol of PLR [25]. The most important issues are: starting the test from the semirecumbent position (adding trunk lowering to leg raising mobilizes venous blood from the splanchnic compartment and magnifies the effects on cardiac preload) and direct measurement of cardiac output at all steps. In our study, PLR was an equivalent of intravenous administration of 250 ml of saline. Change in cardiac output after passive leg raising correlated well with that induced by volume expansion (*p* = 0.000, *r* = 0.822). A 15 % PLR-induced increase of cardiac output predicted the corresponding increase in cardiac output after fluid administration with 79.17 % sensitivity and 81.82 % specificity. These results are in concordance with available literature. Monnet et al. [26] proved that transient hemodynamic changes induced by PLR had an excellent prediction in preload responsiveness in mechanically ventilated ICU patients. In that study, a > 10 % increase of aortic flow induced by PLR was predictive of an increase of aortic flow of >15 % in response to volume expansion. Lamia et al. [27] demonstrated that a PLR-induced increase in stroke volume of ≥12.5 % predicted an increase in stroke volume of ≥15 % after fluid challenge. In the study of Maizel et al. [24], PLR-induced increase of stroke volume/cardiac output by >12 % was highly predictive of volume responsiveness in spontaneously breathing patients. The meta-analysis of 21 studies conducted in 991 adult patients, published recently by Monnet et al. [28], also demonstrated that the changes in CO during a PLR test predicted fluid responsiveness with excellent pooled sensitivity and specificity. The pooled area under the ROC curve in the meta-analysis was 0.95 ± 0.01 [28]. The AUROC in our study (0.805) confirmed very good diagnostic accuracy of the test.

Reliable, real time measurement of stroke volume (implying accurate calculation of cardiac output) remains the key issue in determination of fluid responsiveness [29]. The thermodilution technique has been validated in a variety of experimental models and thermodilution pulmonary artery catheters (PACs) were adopted as the clinical reference standard and subsequently used in the comparison studies [29]. However, the thermodilution devices are invasive, slow-reacting, require stable core temperatures and cannot be used in patients with tricuspid insufficiency. Additionally, there has been observed a decline in PACs use due to the results of prospective, randomized controlled trials assessing the efficacy of PACs in critically ill patients [30–32]. Their results did not justify the widespread use of thermodilution devices in ICU. There are numerous minimally or noninvasive devices available to measure cardiac output in the ICU setting [29]. Bioimpedance, pulse contour analysis, partial rebreathing and pulse wave velocity analysis require technical support and operator experience [29]. Doppler-based echocardiographic techniques are less invasive (transesophageal) or noninvasive (transthoracic), offer real-time beat-to-beat measurements and have similar accuracy as thermodilution pulmonary artery catheters [17, 29]. Despite some technical issues (operator experience, learning curve, accurate ultrasound beam alignment), Doppler echocardiography seems an ideal tool for bedside cardiac output assessment.

## Conclusions

We conclude that dynamic IVC-derived caval index does not predict fluid responsiveness in mechanically ventilated patients with preserved LVEF immediately after elective coronary artery bypass grafting. Passive leg raising is not inferior to intravenous volume expansion in differentiating between fluid responders and nonresponders. Immediate fluid challenge after CABG is safe and well tolerated.

## Abbreviations

AUROC, area under the receiver operator characteristics curve; CABG, coronary artery bypass grafting; CVP, central venous pressure; DBP, diastolic blood pressure; HR, heart rate; ICU, intensive care unit; IVC-CI, inferior vena cava collapsibility index; IVC-DI, inferior vena cava distensibility index; IVC, inferior vena cava; LVEF, left ventricular ejection fraction; LVOT, left ventricular outflow tract; MAP, mean blood pressure; PAC, pulmonary artery catheter; PLR, passive leg raising, PP-pulse pressure; SBP, systolic blood pressure; SD, standard deviation; VTI, velocity-time integral

## Additional file

Additional file 1: IVC_PLR_dataset (XLSX 15 kb)
